# Development of a maize 55 K SNP array with improved genome coverage for molecular breeding

**DOI:** 10.1007/s11032-017-0622-z

**Published:** 2017-02-16

**Authors:** Cheng Xu, Yonghong Ren, Yinqiao Jian, Zifeng Guo, Yan Zhang, Chuanxiao Xie, Junjie Fu, Hongwu Wang, Guoying Wang, Yunbi Xu, Ping Li, Cheng Zou

**Affiliations:** 1 Institute of Crop Science, Chinese Academy of Agricultural Sciences, 12 South Zhongguancun Street, Beijing, 100081 China; 2 CapitalBio Technology Corporation, 18 Life Science Parkway, Beijing, 101111 China; 3 International Maize and Wheat Improvement Center (CIMMYT), El Batan, 56130 Texcoco, CP Mexico; 4 Ministry of Agricultural Scientific Observing and Experimental Station of Maize in Plain Area of Southern Region, Nantong University, 9 Seyuan Road, Nantong, Jiangsu 226019 China; 5 Nantong Xinhe Bio-Technology, 1692 Xinghu Avenue, Nantong, Jiangsu 226019 China

**Keywords:** Maize, Single nucleotide polymorphism (SNP), 55 K SNP array, DNA fingerprinting, Molecular breeding

## Abstract

With the decrease of cost in genotyping, single nucleotide polymorphisms (SNPs) have gained wide acceptance because of their abundance, even distribution throughout the maize (*Zea mays* L.) genome, and suitability for high-throughput analysis. In this study, a maize 55 K SNP array with improved genome coverage for molecular breeding was developed on an Affymetrix® Axiom® platform with 55,229 SNPs evenly distributed across the genome, including 22,278 exonic and 19,425 intronic SNPs. This array contains 451 markers that are associated with 368 known genes and two traits of agronomic importance (drought tolerance and kernel oil biosynthesis), 4067 markers that are not covered by the current reference genome, 734 markers that are differentiated significantly between heterotic groups, and 132 markers that are tags for important transgenic events. To evaluate the performance of 55 K array, we genotyped 593 inbred lines with diverse genetic backgrounds. Compared with the widely-used Illumina® MaizeSNP50 BeadChip, our 55 K array has lower missing and heterozygous rates and more SNPs with lower minor allele frequency (MAF) in tropical maize, facilitating in-depth dissection of rare but possibly valuable variation in tropical germplasm resources. Population structure and genetic diversity analysis revealed that this 55 K array is also quite efficient in resolving heterotic groups and performing fine fingerprinting of germplasm. Therefore, this maize 55 K SNP array is a potentially powerful tool for germplasm evaluation (including germplasm fingerprinting, genetic diversity analysis, and heterotic grouping), marker-assisted breeding, and primary quantitative trait loci (QTL) mapping and genome-wide association study (GWAS) for both tropical and temperate maize.

**Electronic supplementary material:**

The online version of this article (doi:10.1007/s11032-017-0622-z) contains supplementary material, which is available to authorized users.

## Introduction

Over the past 20 years, diverse genetic markers, such as restriction fragment length polymorphism (RFLP), simple sequence repeat (SSR), and single nucleotide polymorphism (SNP), have been adopted for assessing the genetic characteristics of populations or germplasm, mapping quantitative trait loci (QTL), and facilitating the selection of breeding materials that bear desired genes/alleles or haplotypes in both plant genetics and breeding programs. Compared with other genetic markers, SNPs possess several prominent advantages and have become a trendy choice with the development of high-throughput sequencing and array-based technologies. First of all, SNPs, as the ultimate form of genetic markers, are more abundant than other markers throughout the genome. In plants, the SNP density ranges from 6 to 22 SNPs per 1 kb sequence (Shen et al. 2004; Clark et al. 2007; Gore et al. 2009). The number of available SNP markers increases exponentially with large-scale whole genome or transcriptome sequencing. For example, in maize HapMap 3, 83 million SNPs and InDels have been identified based on 1218 maize germplasms (Bukowski et al. 2015). Secondly, SNPs are a kind of “biallelic marker,” which makes it much easier to integrate and compare sequencing data from different sources. As the expense and labor costs for genotyping SNPs have decreased dramatically, a variety of SNP genotyping platforms has been widely applied to genetic research and breeding programs. These include multiplex chip-based SNP detection, uniplex SNP genotyping, and sequencing. When genotyping requires a small number of markers and a large number of samples, uniplex SNP genotyping platforms, such as Kompetitive Allele Specific PCR (KASP), may be more suitable. However, high marker density is indispensable when conducting high-resolution fingerprinting, large-scale QTL mapping, genome-wide association studies (GWAS), and genome selection (GS). In this case, high-throughput chip-based and sequencing-based platforms are preferred to uniplex SNP genotyping platforms. With the rapid innovation of sequencing technology, sequencing-based platforms are playing an increasingly important role in genotyping (Romay et al. 2013; Zhang et al. 2015; Wu et al. 2016). Ideally, whole genome sequencing could identify all of the SNPs across the genome; however, the high cost is still a barrier. Lower cost alternatives, restriction site-associated DNA sequencing (RADseq) (Baird et al. 2008) and genotyping by sequencing (GBS) (Elshire et al. 2011; Poland et al. 2012; Glaubitz et al. 2014), can also identify more than one million SNPs with imputation (Glaubitz et al. 2014). Although RADseq and GBS have emerged as promising choices for an efficient and less costly genotyping strategy, missing data and low coverage are two main flaws which restrict their application. Firstly, mutations at a restriction enzyme recognition site will lead to a failure to detect SNPs in that region. Secondly, the low sequencing coverage will result in a high rate of false positives, especially for species with larger and more complex genomes. However, for chip-based platforms, the SNP design is more targeted, and SNPs are more likely to be distributed in gene regions associated with agronomic traits. Thus, the stability and reliability of chip-based platforms are much higher, allowing more convenient data comparing and sharing across laboratories and experiments worldwide. In addition, chip-based platforms with a flexible number of SNPs varying from several thousand to one million can also be used to perform multiplex genotyping, which allows simultaneous genotyping of as many as 384 samples.

Maize is a widely cultivated crop serving as an important staple food and livestock feed. It is also a species with tremendous genetic diversity. Based on whole genome sequencing and transcriptome analysis on worldwide germplasm collections, more than 83 million SNPs and InDels have been identified according to B73 reference (Chia et al. 2012; Fu et al. 2013; Bukowski et al. 2015). These SNPs can be used to design arrays/chips for genotyping. Several high-throughput genotyping platforms have been established in maize using Illumina® Beadchip and Affymetrix® Axiom®. The earliest Illumina® 1536 SNP chip was developed in 2009 (Yan et al. 2010; Lu et al. 2009). Soon after that, TraitGenetics, INRA, and Syngenta published Illumina® MaizeSNP50 Beadchip. Recently, high-density arrays have been designed for maize, namely the 600 K Affymetrix® Axiom® Maize Genotyping Array, or MaizeSNP600K (Unterseer et al. 2014). These arrays/chips have been widely used for characterizing genetic properties of diverse maize populations, fingerprinting important germplasms (Lorenz and Hoegemeyer 2013; Tian et al. 2015), performing GWAS, and QTL mapping (Cook et al. 2012; Li et al. 2013).

However, there are still several challenges in using existing genotyping arrays. Firstly, currently available SNP chips were developed based on the B73 reference genome, whereas previous studies have demonstrated that single-genome based references provide only partial genome coverage for a crop species (Bukowski et al. 2015; Lu et al. 2015; Hirsch et al. 2014), especially for maize, which has rich genetic diversity. Overdependence on the B73 reference genome may lead to a failure in map-based cloning, missing of important QTL/genes in genetic and association mapping, biased estimation of genetic diversity, population structure, linkage disequilibrium (LD), identity by descent (IBD) and haplotypes, and inefficiency and unpredictable results in marker-assisted selection. However, tropical maize germplasms originating from Mexico host richer genetic diversity and are a better resource for abiotic and biotic stress tolerance. Secondly, rare genes, which may play a key role in major breeding breakthroughs, are mostly precluded. Thirdly, SNP chips applied in modern breeding programs usually have low genome coverage and uneven distribution across chromosomes, resulting in loss of available genetic information. Finally, an insufficient number of SNPs related to known genes are included in current SNP chips. Therefore, this study aims to develop an unbiased genotyping array which can capture the variants both in temperate and tropical germplasms. We printed a total of 55,229 probes on Affymetrix® Axiom® Maize Genotyping Array. To demonstrate the utility of this array in genotyping both temperate and tropical lines, we tested 593 inbred lines with diverse genetic backgrounds. We found that this 55 K array is efficient in resolving heterotic groups and is potentially suitable for germplasm fingerprinting, QTL mapping, and GWAS in both tropical and temperate maize populations.

## Materials and methods

### Design of the Maize 55 K SNP Affymetrix® Axiom® Genotyping Array

To build a reliable and balanced genotyping array, we selected SNPs from diverse resources (Supplementary Table S1). Firstly, 30,133 SNPs were selected from the 600 K Affymetrix® Axiom® Maize Genotyping Array. These SNPs were selected to ensure even distribution across the genome, with at least one SNP per 100 kb. Next, we collected SNP data of 1090 tropical and temperate elite lines genotyped using the Illumina® MaizeSNP50 BeadChip, from which 4049 SNPs with a missing rate less than 5% and a high level of polymorphism in both tropical and temperate maize were selected.

In a previous study (Fu et al. 2013), a million highly accurate SNPs were identified using RNA-seq data from 368 maize lines with nearly equal proportions of tropical and temperate germplasm. These SNPs were highly concordant with the genotyping results from the MaizeSNP50 BeadChip and the Sequenom MassArray iPLEX genotyping system. Therefore, 9395 SNPs, which have maximum fixation index (*F*_*ST*_) among tropical and temperate populations, were selected. These SNPs are all located in the genic regions of the maize genome. It has been shown that gene density in maize is correlated with recombination rate, and thus including the genetic variation located in genes will increase the genetic information for regions of high recombination rate as well. High SNP density in high recombination rate regions will improve the resolution in linkage analysis and GWAS.

Maize is a species with a high level of nucleotide polymorphism. We have detected many dispensable sequences (defined as sequences that exist in only a subset of individuals) based on resequencing 79 tropical and temperate inbred lines. A total of 4067 SNPs were selected based on the dispensable genome (defined as the part of the genome that exists in only a subset of individuals) (unpublished data) to capture the unique genetic diversity existing in tropical maize. A great number of genes that are related to maize development and desirable agronomic traits have been extensively studied. To cover these functional genetic loci, we included all available SNPs from 368 known genes in maize (https://genomevolution.org/wiki/index.php/Classical_Maize_Genes). As transgenic breeding technology blossoms, more and more transgenic crops will be produced in the near future. Molecular markers should be explored as a powerful tool for facilitating the transformation process and detecting transgenic plants. Therefore, we also included 132 SNPs that are tags for published transgenic events on the maize 55 K SNP array.

After pooling all candidate SNPs, LD pruning was performed to avoid including redundant information derived from neighboring SNPs in the same haplotype. As a result, a total of 55,229 SNPs were printed on the 55 K array. According to the sequence annotation results with the B73 filtered gene set, 22,278 and 19,425 SNPs were distributed in the exonic and intronic regions, respectively (Supplementary Table S2). Information about 55 K SNP variants distribution and association with known genes and heterosis groups is provided in Supplementary Table S3 and Supplementary Fig. S1.

### Plant materials and DNA extraction

To evaluate the performance of 55 K array, we genotyped 593 diverse maize inbred lines representing temperate, tropical, and subtropical germplasm from China (308), the USA (96), and CIMMYT (189) were genotyped. Among them, the lines from China and the USA are mainly public inbred lines including 55 newly developed elite lines. The collection from CIMMYT is a widely used germplasm in the tropical regions. Detailed information about the 593 inbred lines is listed in Supplementary Table S4.

For each line, 15 seeds were grown in one pot and DNA was extracted from five uniform seedlings representing the inbred line with cetyltrimethylammonium bromide (CTAB) method (Saghai-Maroof et al. 1984) or TIANGEN plant genomic DNA kit. Among 593 tested lines, H201, Ji046, Ji495, K14, and B73 were genotyped twice as biological replications.

### Evaluation of the maize 55 K SNP array using a diverse set of 593 inbred lines

For each inbred line, 200 ng genomic DNA was used for genotyping on the Affymetrix® GeneTitan® platform with the Axiom® myDesign GW genotyping array according to manufacturer’s protocol. The 593 maize samples were distributed in three 384-well plates with other maize materials. The raw signal CEL files were processed using the Axiom® genotyping best practices. All three plates passed Dish QC. On these three plates, 96, 100, and 99% samples passed dQC with QC criteria >97%, respectively. The probe QC was then determined using samples passing the QC and variants were classified into six major categories, “PolyHighResolution” (featured by fulfilling all cluster metric criteria with at least two examples of the minor allele and generally recognized as the polymorphic SNPs with good quality), “MonoHighResolution” (featured by good cluster resolution with less than two examples of the minor allele), “NoMinorHomozygote” (featured by two clusters with no examples of the minor homozygous genotypes), “OffTargetVariant” (where an off target variant cluster is called), “CallRateBelowThreshold” (featured by SNP call rate below threshold, but the other properties of cluster are above threshold), and “Other” (where more than one cluster properties are below threshold) (Gao et al. 2014). We found that the number of “PolyHighResolution” that was the most desired and stringent class of variants, increased by 16% after applying inbred correction when genotyping inbred lines (Supplementary Table S5). Therefore, parameter 14 was used in our calculation, which allows some level of heterozygosity.

### Population structure and genetic diversity analysis

Population structure analysis was implemented with a non-parametric approach by fastSTRUCTURE (Raj et al. 2014), a variational Bayesian framework, using SNPs (MAF > 0.05). Different numbers of ancestral clusters (*k* = 2 to 12) were tested continuously with a default convergence criterion 20 times. The results from replicate runs came into integration by CLUMPP program with full search method.

The phylogenetic tree was constructed with an integrated pipeline called SNPhylo (Lee et al. 2014). To decrease the running time, we reduced the SNPs density by LD-based pruning. A total of 7499 SNPs passed the threshold (no SNP pairs with *r* ^2^ larger than 0.1 in a 50 SNP window). The remaining SNPs were aligned with MUSCLE, and the maximum likelihood tree was calculated by DNAML implemented in SNPhylo. Polymorphism information content (PIC) value (Botstein et al. 1980), describing the relative value of each marker on the amount of polymorphism exhibited was calculated as

$$ \mathrm{PIC}=1-\left(\sum_{i=1}^n{P}_i^2\right)-\sum_{i=1}^{n-1}\sum_{j= i+1}^n2{P}_{\mathrm{i}}^2{P}_{\mathrm{j}}^{2.} $$

where *P*_i_ and *P*_j_ are the population frequency of the *i*th and the *j*th allele.

We used the average nucleotide difference to evaluate the genetic distance between samples, which is one minus the average of identical by state (IBS). IBS was calculated using PLINK 1.07. Genomic divergence between different populations and pairwise nucleotide diversity within a population were calculated using VCFtools version 0.1.12.0 (Weir and Cockerham 1984).

### Analysis of relative kinship and linkage disequilibrium decay

A total of 39,296 SNPs with MAF > 0.05 and missing data <20% were used for estimating the relative kinship of 593 inbred lines with the software TASSEL 5.2.23 (Bradbury et al. 2007). A value close to 0 indicates a weaker relationship between two random inbred lines, while a value close to 1 indicates a stronger relationship. Average LD between SNPs within the tropical lines, temperate lines, and the entire panel, were also calculated in TASSEL 5.2.23 using all the SNPs with MAF greater than 0.05. A 50 kb sliding window was selected and squared Pearson correlation (*r* ^2^) was used to evaluate the level of LD decay.

### Development of a core SNP set from the 55 K array for simplified fingerprinting and germplasm evaluation

Based on our genotyping for 593 diverse maize inbred lines, we further selected 1000 SNP markers as a core set for the simplified fingerprinting experiment. We intended to include probes which can represent diverse polymorphism patterns and be evenly distributed on ten chromosomes. Firstly, we classified 55 K SNPs into 1000 clusters based on their genotyping patterns in 593 tested lines (defined as genotyping bins). Secondly, the maize genome was divided into 2000 bins (defined as genetic bins). Then, we applied a genetic algorithm to identify the best sets of probes. We picked up a start set with one random probe in each genotyping bin, and the mutation was made to maximize the number of probes which located in different genetic bins. We picked two independent 1000 SNPs sets which start with two random sets of probes (Supplementary Table S6). The final sets contain probes that can cover 90% of the genotyping bins and genetic bins.

## Results

### Design features of the maize 55 K SNP array

To test the reproducibility of our design, concordant rates between five pairs of two biological replications were verified. A high level of concordant genotype calls in the range of 97.1 to 98.9% was observed (Supplementary Table S7). The missing rate per sample based ranged from 0.33 to 5.17%, with an average of 1.83%, indicating that majority of our probes are located in the core genome (defined as the part of the genome that exists in all individuals) (Hirsch et al. 2014) (Supplementary Table S4). There is a positive correlation between the missing rate and the heterozygous rate. We also compared the missing rates for 30 randomly selected samples whose DNA were isolated using two different methods, CTAB and a commercial kit, to see if the platform is sensitive to DNA quality. On average, no significant difference was found (Supplementary Table S8; unpaired *t* test, df = 58, *P* value >0.21).

**Fig. 1:**
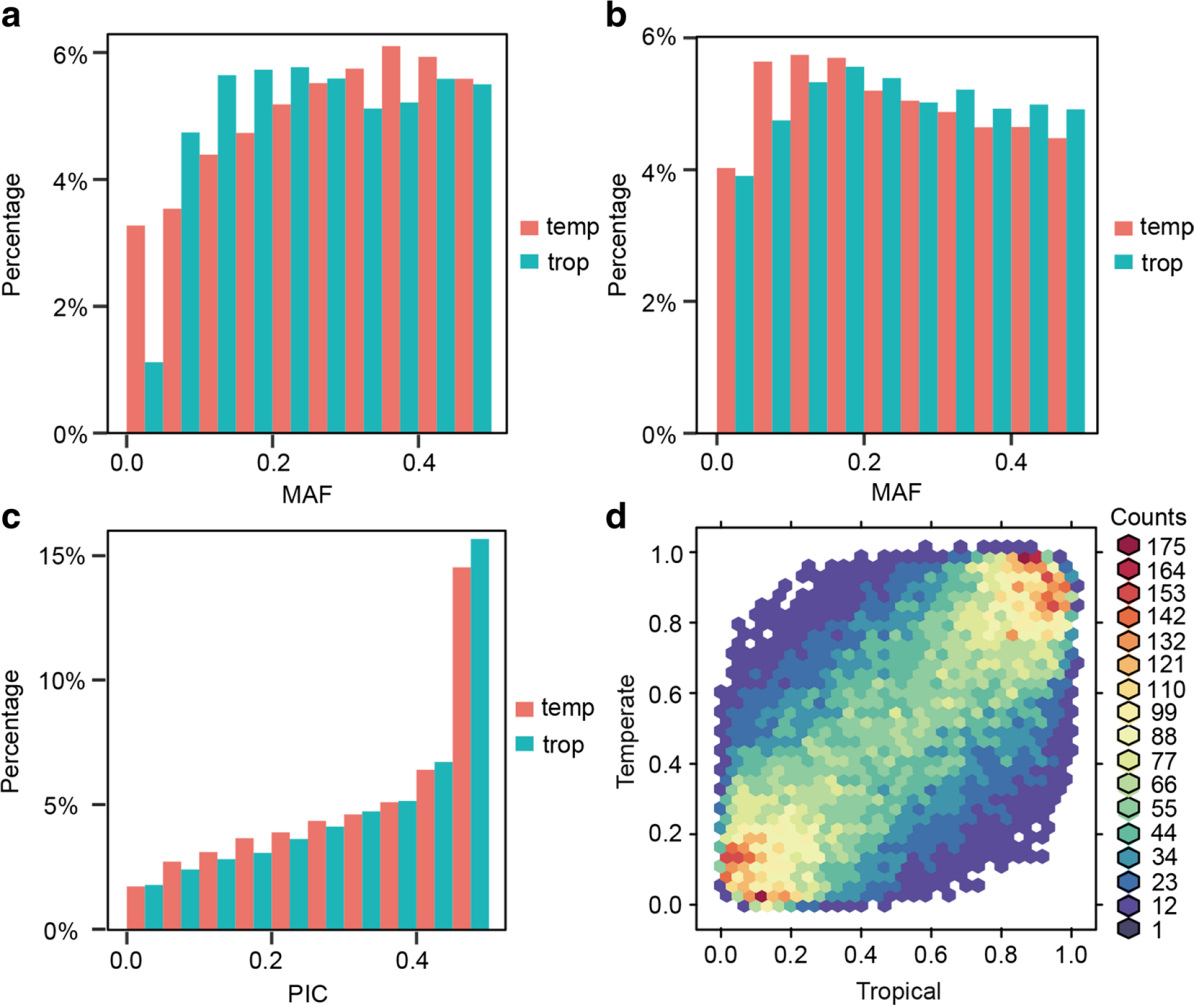
Balanced array design for temperate and tropical population. **a** Frequency distribution of minor allele frequency for Illumina® MaizeSNP50 Beadchip based on 279 temperate (tem) lines and 282 tropical (trop) inbred lines. **b** Frequency distribution of minor allele frequency for maize 55 K SNP array based on 255 temperate lines and 262 tropical inbred lines. **c** Polymorphism information content (PIC) score for temperate and tropical germplasms. **d** Two-dimensional folded site frequency spectra for SNPs in tropical (*x* axis) and temperate (*y* axis) maize inbred lines. The number of SNPs detected is color-coded according to the color scale plotted on the right

To determine if our array design is biased towards tropical or temperate germplasms, PIC, MAF, and two-dimensional folded site frequency spectra (SFS) were examined (Fig. 1b, c, d). No significant difference in PIC or MAF was found between tropical and temperate germplasms (Wilcoxon test, *P* value > 0.05). The unbiased design was further validated in the two-dimensional folded SFS. The most enriched pattern was derived from alleles with similar frequency in tropical and temperate germplasm (Fig. 1d). A total of 51 SNPs with high allele frequency differentiation (AFD) between temperate and tropical maize (AFD > 0.660) were identified, and these SNPs are distributed across the entire genome. Within the region (upstream 5 kb and downstream 5 kb) of the significant SNPs, several genes of interest were detected (Table 1). Of them, GRMZM2G336824, GRMZM2G367023, GRMZM2G003318, GRMZM5G831712, GRMZM2G035068, and GRMZM2G095786 are related to the response of plants to a series of stresses and diseases (Vincent et al. 2005; Wang et al. 2004; Jones et al. 2006; Adams-Phillips et al. 2010; Huang et al. 2016; Navarro et al. 2006). Another four genes are associated with plant developmental regulation, including nutrient translocation in developing seeds, cell cycle regulation, and regulation of signal transduction (Rodriguez 1998; Tomaštíková et al. 2015; Mertens et al. 1990; Zuber et al. 2010). In particular, the differentiation of GRMZM2G048313 (related to photosynthesis and respiration) and GRMZM2G040033 (related to phosphoenolpyruvate-specific transport) (Balk and Lobréaux 2005; Knappe et al. 2003) between tropical and temperate maize may have resulted from post-domestication selection, which has transformed wild maize into cultivated types that have adapted to diverse environments different from the ancestral location.

**Table 1:** Genes located within 10 k interval of SNPs with large allele frequency differentiation between temperate and tropical maize

Gene id	Chr	Pos	Tem	Tro	AFD	Annotation	Biological process involved in
GRMZM2G336824	1	66,402,581	0.886	0.124	0.762	O-methyltransferase family	Response to stress
GRMZM2G367023	1	98,327,770	0.267	0.946	0.679	DNAJ heat shock domain containing protein	Response to stress
GRMZM2G003318	1	101,278,790	0.905	0.138	0.767	PAP fibrillin family domain containing protein	Resistance to biotic and abiotic stresses
GRMZM2G479665	2	14,626,634	0.118	0.840	0.722	Protein phosphatase 2C family protein	Regulators of signal transduction pathways
GRMZM5G831712	2	158,103,580	0.136	0.845	0.709	ADP-ribose polymerase 2	Response to biotic stress
GRMZM2G433150	2	173,189,785	0.849	0.147	0.702	Targeting protein for Xklp2 protein family	Cell cycle regulation and signaling transduction
GRMZM2G035068	4	206,842,424	0.933	0.219	0.714	DEAD box RNA helicase family protein	Response to stress
GRMZM2G048313	6	1,369,191	0.147	0.816	0.669	Iron-sulfur cluster	Photosynthesis, respiration and nitrogen fixation
GRMZM2G040033	6	1,538,822	0.052	0.720	0.668	Phosphoenolpyruvate translocator protein	Phosphoenolpyruvate-specific transport
AC194439.3_FG002	8	84,948,532	0.875	0.141	0.734	Fructose 6-phosphate 1-phosphotransferase	Seeds development
GRMZM2G444801	9	20,350,849	0.883	0.139	0.744	Sulfate transporter	Sulfate translocation within developing seeds
GRMZM2G095786	9	150,017,086	0.052	0.713	0.662	Auxin signaling F-box 2	Response to stress

Allele frequency differentiation was calculated by absolute values of temperate value subtracting tropical value. *Chr* chromosome, *Pos* position, *Tem* temperate, *Tro* tropical, *AFD* allele frequency differentiation

### Population structure and genetic diversity

**Fig. 2:**
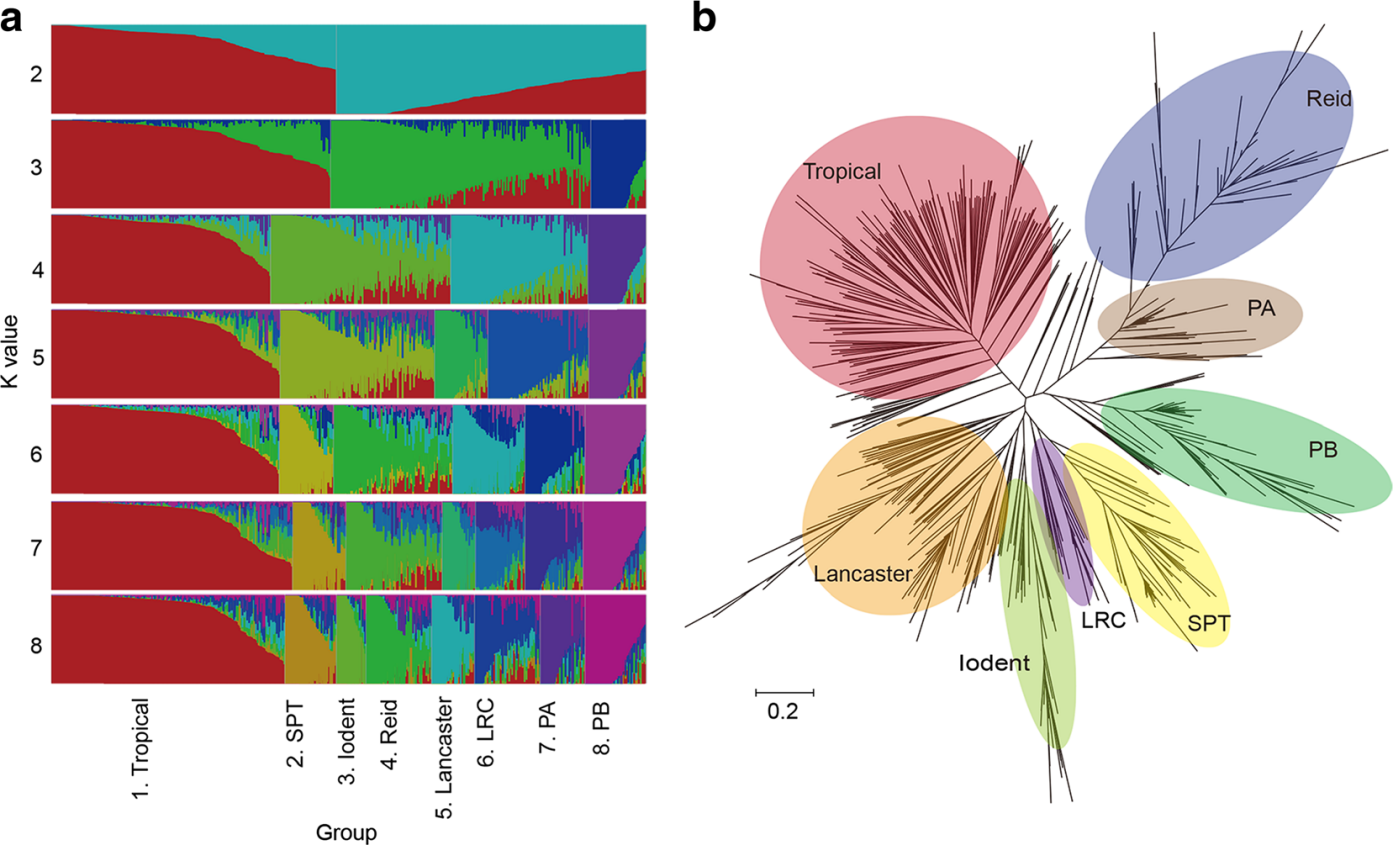
**a** Inferred population structure for 593 diverse *maize inbred lines* with different total number of populations (*k* = 2 to 8). Each *vertical line* represents one sample. Membership coefficients (Q values) were summarized from 20 replications performed by fastSTRUCTURE. **b** Maximum likelihood phylogenetic tree for 593 *maize inbred lines*. Major population groups were determined by population structure

The identification of population substructure is essential for quantitative genetic analysis, population genetics and utilization of heterosis. To test the capacity of our array to detect population structure, an admixture-model analysis of 593 representative inbred lines with high diversity was implemented in fastSTRUCTURE with 20 runs for *k* from 2 to 12. The results indicate that when *k* = 2, two major groups, representing temperate and tropical ones could be claimed. Compared with the pedigrees of the tested lines, however, the model-based groups were largely consistent with known pedigrees when *k* = 8, so that the 593 tested lines could be largely divided into eight groups (Fig. 2a). Six of these groups (Groups 2, 4, 5, 6, 7, 8) include most of Chinese and US temperate inbred lines and correspond to the six major Chinese heterotic groups, SPT, Reid, Lancaster, LRC, PA, and PB. Group 2, SPT, originating in China, includes widely-used inbred lines Huangzao4 and Chang7-2. Group 4, Reid, consists of two typical Reid-type lines, B73 and B84. Group 5, Lancaster, represents the majority of Chinese inbred lines closely related to Mo17. Group 6, LRC, is also from China, including representative lines Dan340 and Dan360. Group 7 is inferred as PA, including Ye478, which is derived from a US commercial hybrid. Group 8 belongs to PB heterotic group, consisting of representative inbred lines Qi319 and 178, most of which were derived from the US elite hybrid P78599. Another large group, Group 1, includes lines mostly from CIMMYT as well as southwestern China representing tropical/subtropical germplasm, such as CML202 and Silunuo. Group 3 with 30 inbred lines mainly collected from the USA is derived from the widespread Iodent germplasm, which could not be combined with any of the groups above. In conclusion, the division of the subgroup is approximately consistent with previous researches, indicating that the maize 55 K SNP array has a strong capacity to distinguish the existing maize heterotic groups from each other with high resolution.

To obtain detailed information about the genetic diversity among different groups of sequenced maize lines, a maximum likelihood tree was constructed with SNPhylo (Fig. 2b). The 593 tested lines were evidently divided into three branches, which are consistent with previous researches, inferred as SS, NSS, and tropical/subtropical. When classified into eight branches, the result is highly consistent with the fastSTRUCTURE results. Among eight subgroups, LRC and Iodent groups were located in the intermediate zone of heterotic groups, which corresponds to the empirical breeding experience. Iodent, the prominent branch consisting of inbred lines PHG29 and PHJ90, was positioned between typical SS and NSS groups as expected based on the heterotic pattern and combining ability model that classify the Iodent germplasm into NSS group currently. As a basic breeding population, the US Reid (BSSS) group has provided abundant germplasm for maize breeding in China. The tree also shows that PA group (also known as “improved Reid” in China), consisting of a core inbred line Ye478, had a quite close relationship with Reid (BSSS). In summary, the phylogeny tree constructed using our array data is highly consistent with existing heterotic groups and known pedigrees.

The genetic diversity among different groups, which was an important index in evaluating array performance, was further examined using average nucleotide difference. When six to ten typical lines were chosen to calculate the pairwise nucleotide difference (Supplementary Table S9), the average nucleotide difference within one group ranged from 18.0 (Iodent group) to 31.3% (tropical group). For pairs from different groups, the average nucleotide differences ranged from 33.3 (between PA and Reid groups) to 40.2% (between SPT and Reid groups). In summary, the SNP markers included in the 55 K array detected a higher level of genetic diversity compared with previous estimates (Lu et al. 2009) and revealed consistent results with breeding practices as well as known pedigrees (Table 2).

**Table 2:** Pairwise differences between eight groups. The lower diagonal indicates the average genetic distance between groups. The diagonal cells are pairwise nucleotide diversity within group

Group	SPT	LRC	Lancaster	Reid	PB	PA	Iodent	Tropical
SPT	0.219							
LRC	0.369	0.253						
Lancaster	0.388	0.368	0.193					
Reid	0.403	0.380	0.391	0.238				
PB	0.381	0.373	0.370	0.376	0.199			
PA	0.380	0.364	0.360	0.333	0.345	0.242		
Iodent	0.399	0.381	0.386	0.368	0.382	0.375	0.181	
Tropical	0.368	0.374	0.396	0.396	0.357	0.373	0.387	0.314

### Relative kinship and LD decay

To determine the degree of relatedness between inbred lines, we calculated relative kinship for 593 inbred lines based on 39,296 SNPs. The distribution of relative kinship values shows that 59% of the values range from 0 to 0.05 and 25% range from 0.05 to 0.2 (Supplementary Fig. S2). This result indicates that most of the 593 inbred lines are distantly related to each other.

Mean *r* ^2^ values were pooled over all chromosomes for temperate, tropical, and entire panels with average distance. The average distance of LD decay with *r* ^2^ = 0.1 was 100–150 kb, 400–450 kb, and 200–250 kb, for the tropical, temperate, and entire panel, respectively (Supplementary Fig. S3). The average LD distance in the temperate panel was 2.5 to 4.5 times larger than in the tropical panel, which is basically consistent with previous reports (Yan et al. 2009; Lu et al. 2011). The average distance of LD decay computed in our study was much larger than in the previous study with high marker density (Wu et al. 2016). This difference can be attributed to the low marker density in our genotyping array. In order to increase the marker coverage in the whole genome, we only allow few SNPs in every 100 kb. And we have removed adjacent SNPs on purpose. Compared to the studies with similar marker density as us, the LD level of 593 lines is consistent with the results of 367 elite lines with an average LD distance of 391 kb (Wu et al. 2014) and 500 kb of 285 dent inbred lines (Riedelsheimer et al. 2012). This 55 K SNP array can be used to get a rough estimation of LD, and more markers will be needed to get a more accurate LD estimation.

### Comparison with the existing Illumina® MaizeSNP50 BeadChip

Illumina® MaizeSNP50 BeadChip, which is composed of 49,585 markers, has been extensively used in genetic mapping and diversity analysis. We compared the performance of our 55 K array with the existing BeadChip. Firstly, missing is high for the existing BeadChip when the results from different experimental batches were combined, which is due to the random loss of approximately 10% of the beads with each run. For example, in the maize germplasms that have been genotyped with the BeadChip in three different labs, only 31,755 SNPs were common (shared). This makes it difficult to compare and integrate samples from different batches (Supplementary Fig. S4). To be more comparable, missing and heterozygous rates between the existing BeadChip and our array were compared by randomly selecting the same set of 140 inbred lines (70 tropical and 70 temperate lines). The average missing rate for the BeadChip was 3.6%, whereas it was approximately 1.9% with our array. The average heterozygous rate was 1.8 and 0.7% for BeadChip and our array, respectively. Secondly, for 70 tropical lines, missing and heterozygous rates with the BeadChip were 5.8 and 3.2%, respectively, which were much higher than those from our 55 K array (2.3 and 0.7%, respectively). Thirdly, the MAFs in tropical and temperate germplasm were compared (Fig. 1a, b). For the 55 K array, tropical germplam had more probes with lower MAF than BeadChip, which is due to our intended selection for SNP markers to have a better coverage of genetic variation in the tropical maize. However, when the average nucleotide differences between samples were compared, no significant differences were found between these two arrays.

### Features of two core-sets of 1000 SNP markers

**Fig. 3:**
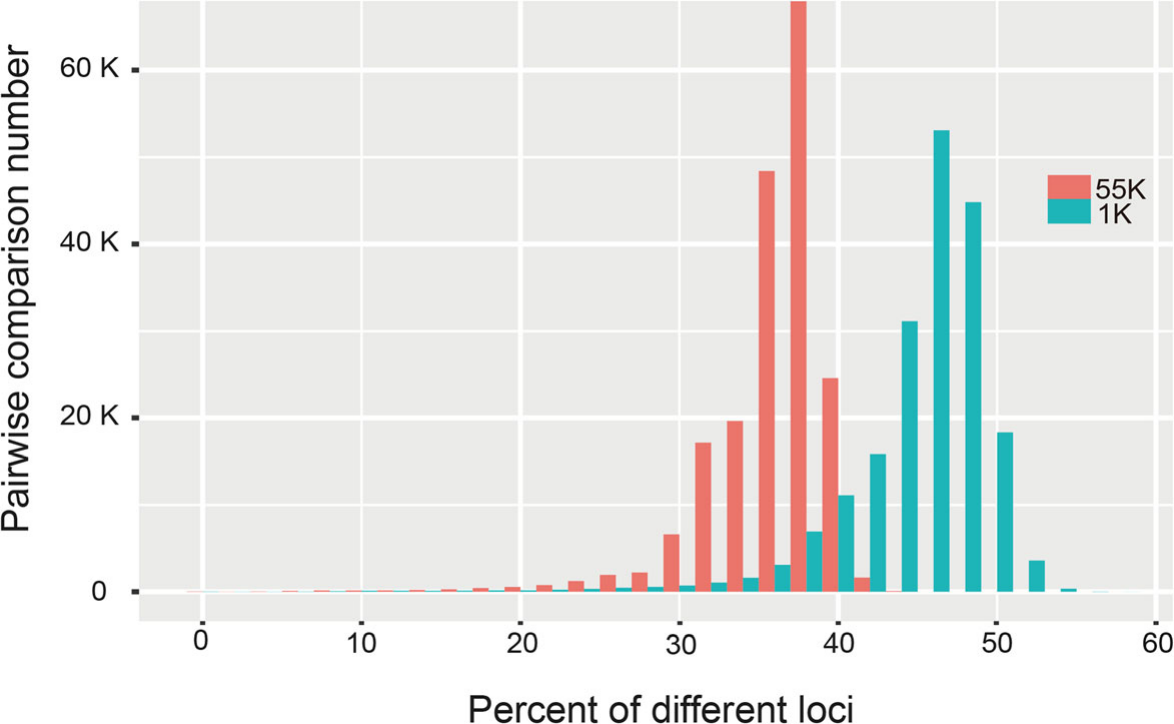
Distribution of different locus percentages by pairwise comparative analysis of 593 samples based on 1000 SNPs and 55 K array

In many cases, considering the balance between cost and information content, core probes containing fewer SNP markers are preferred in fingerprinting and germplasm evaluation. Therefore, we adopted a genetic algorithm to identify two sets of 1000 representative SNPs based on the 593 diverse maize inbred lines we genotyped. We compared the performance of the 1000 SNP sets and the maize 55 K SNP array using the 593 samples evaluated in this study (Fig. 3). Average differential locus rates among inbreds are approximately 45% (median of 46%) and 36% (median of 37%) for both the two sets of 1000 SNPs and 55 K array, respectively.

## Discussion

### The importance of a maize genotyping array with improved genome coverage

Compared with sequencing-based genotyping, as a high-density marker genotyping platform, chip-based genotyping has several advantages. The platform is high-throughput and automatic. The procedure for data generation and analysis can be performed with a standard protocol, and the output is highly comparable across assays, people, machines, and laboratories. As a result, genotypic information can be generated, integrated, and shared easily. This is important for long-term research and breeding programs.

SNP chip design is a challenging task because many factors need to be balanced. Firstly, the number of markers determines the marker density in the chip, and thus the genetic mapping resolution and the power of diversity analysis. However, the more SNPs are printed on, the higher cost per sample will be for genotyping. For germplasm evaluation and rough mapping of genes and QTL, several thousands of markers will be enough (Tian et al. 2015). But for precision association mapping, a marker density of one marker per 100–200 bp is suggested for maize due to its fast LD decay (Beissinger et al. 2013).

Secondly, SNPs with higher MAF are usually preferred in chip designs in order to increase the average allelic differentiation. However, MAF is not a fixed value for different sources of germplasm. Some SNPs that have been identified with MAF > 0.2 in a given sample may vary a great deal in other samples. As genetic diversity is largely represented by SNPs with MAF, eliminating low MAF SNPs from chip design will produce biased results when the chip is used for diverse germplasms. Considering that many important traits in diverse germplasms may be associated with low MAF SNPs, including some of the low MAF sites in the design, like we did for the 55 K array, will facilitate diversity analysis and genetic mapping.

Thirdly, maize is a species with a great amount of genetic variation and a large number of structural variations (SVs) across the genome. If a probe located in regions with SVs, it might introduce artificial missing or heterozygote calls in the genotyping. In our analysis, the missing rate for probes was correlated with heterozygous rate, suggesting that these SNPs might be located in the dispensable genome. As more genotyping results are accumulated, we should be able to pick probes that are more likely located in the core genome.

Fourthly, single reference genomes only represent a portion of the genetic variation for a specific crop species, and the development of pan-genomes (defined as the sum of the core genome and the dispensable genome) (Hirsch et al. 2014) and markers with improved genome coverage will facilitate gene mapping and MAS for diverse sources of germplasm, for example, tropical germplasms in maize. In this study, 4067 SNP markers that are not covered by the current maize reference genome were selected to improve the genome coverage.

### Applications of the maize 55 K SNP array

The current 55 K SNP array was designed to have several important properties, which make it unique in maize germplasm evaluation, genetic mapping, and breeding, by including 451 markers that are associated with known genes and two traits of agronomic importance (which is based on personal communications), 4067 markers that are not covered by the current reference genome, 734 markers that are differentiated significantly among heterotic groups, and 132 markers that are tags for important transgenic events (Supplementary Table S10).

As a cross-pollinated crop, the population structure and kinship of maize is more complex than rice and wheat. Since introduced in the 1500s, Chinese maize germplasm has been collected from diverse regions, mainly from the USA and CIMMYT. Our results, which are strongly consistent with empirical pedigrees, have provided detailed information about different subgroups that will be helpful for improving the breeding efficiency and application of heterotic patterns. Our results also indicate that the 55 K array can be applied for fingerprinting of germplasms, classifying heterotic groups, and selection of inbred lines derived from specific heterotic groups.

Many SNPs missing in the B73 reference genome sequence are also included in the maize 55 K SNP array, facilitating fine comparisons of maize germplasms, and protection of intellectual property. Also, owing to the specific SNPs collected from resequencing data (unpublished) as well as RNA-seq data for tropical and temperate maize inbreds with great diversity, the maize 55 K SNP array can be used in primary GWAS and genetic mapping for both tropical and temperate maize germplasms, although markers with higher density are still needed for gene cloning.

Compared with the currently primary marker system, SSRs, the maize 55 K SNP array contains numerous SNPs directly related to the crucial agronomic and quality traits and is more suitable for backcrossing breeding, recurrent selection, gene pyramiding, and genomic selection.

Taking advantage of the long-service life and flexibly replaceable traits of Affymetrix platform, 1–3 K probes on the maize 55 K SNP array are reserved for a personalized design, which could be used to include the key markers for fingerprinting crucial genetic and breeding materials, novel gene identification and variety protection.

## Electronic supplementary material

Supplementary Fig. S1 Distribution of 55 K variants on ten chromosomes. Window size is 1 Mbp. (JPEG 8 kb)

High Resolution (TIFF 3321 kb)

Supplementary Fig. S2 Distribution of pairwise relative kinship values for 593 maize inbred lines. (JPEG 8 kb)

High Resolution (TIFF 10884 kb)

Supplementary Fig. S3 Mean r2 of different physical distances for temperate, tropical and entire panels.(JPEG 10 kb)

High Resolution (TIFF 8715 kb)

Supplementary Fig. S4 Venn diagram summarizing the probe sets that were common in three independent runs of Illumina® MaizeSNP50 BeadChip. (JPEG 15 kb)

High Resolution (TIFF 13268 kb)

Supplementary Table S1 (XLSX 9 kb)

Supplementary Table S2 (XLSX 10 kb)

Supplementary Table S3 (XLSX 9 kb)

Supplementary Table S4 (XLSX 51 kb)

Supplementary Table S5 (XLSX 9 kb)

Supplementary Table S6 (XLSX 32 kb)

Supplementary Table S7 (XLSX 9 kb)

Supplementary Table S8 (XLSX 9 kb)

Supplementary Table S9 (XLSX 10 kb)

Supplementary Table S10 (XLSX 14 kb)
