# Supplementary figures and images for: Development of a maize 55 K SNP array with improved genome coverage for molecular breeding
Source: Mol Breed. 2017 Feb 16;37(3):20. doi: 10.1007/s11032-017-0622-z (PMC5311085; doi:10.1007/s11032-017-0622-z)

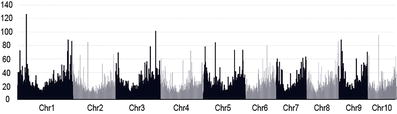

Supplement: Supplementary file 1 — Distribution of 55 K variants on ten chromosomes. Window size is 1 Mbp. (JPEG 8 kb) [file 11032_2017_622_Fig4_ESM.jpg]

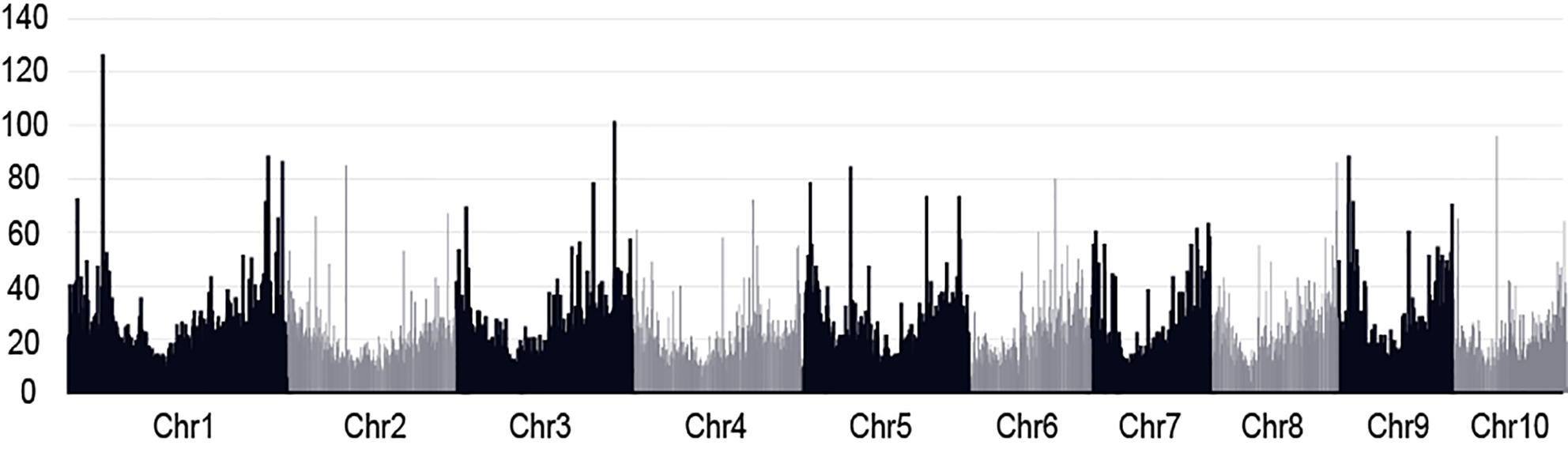

Supplement: Supplementary file 2 — High Resolution (TIFF 3321 kb) [file 11032_2017_622_MOESM1_ESM.tif]

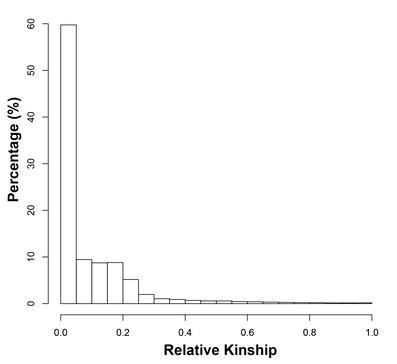

Supplement: Supplementary file 3 — Distribution of pairwise relative kinship values for 593 maize inbred lines. (JPEG 8 kb) [file 11032_2017_622_Fig5_ESM.jpg]

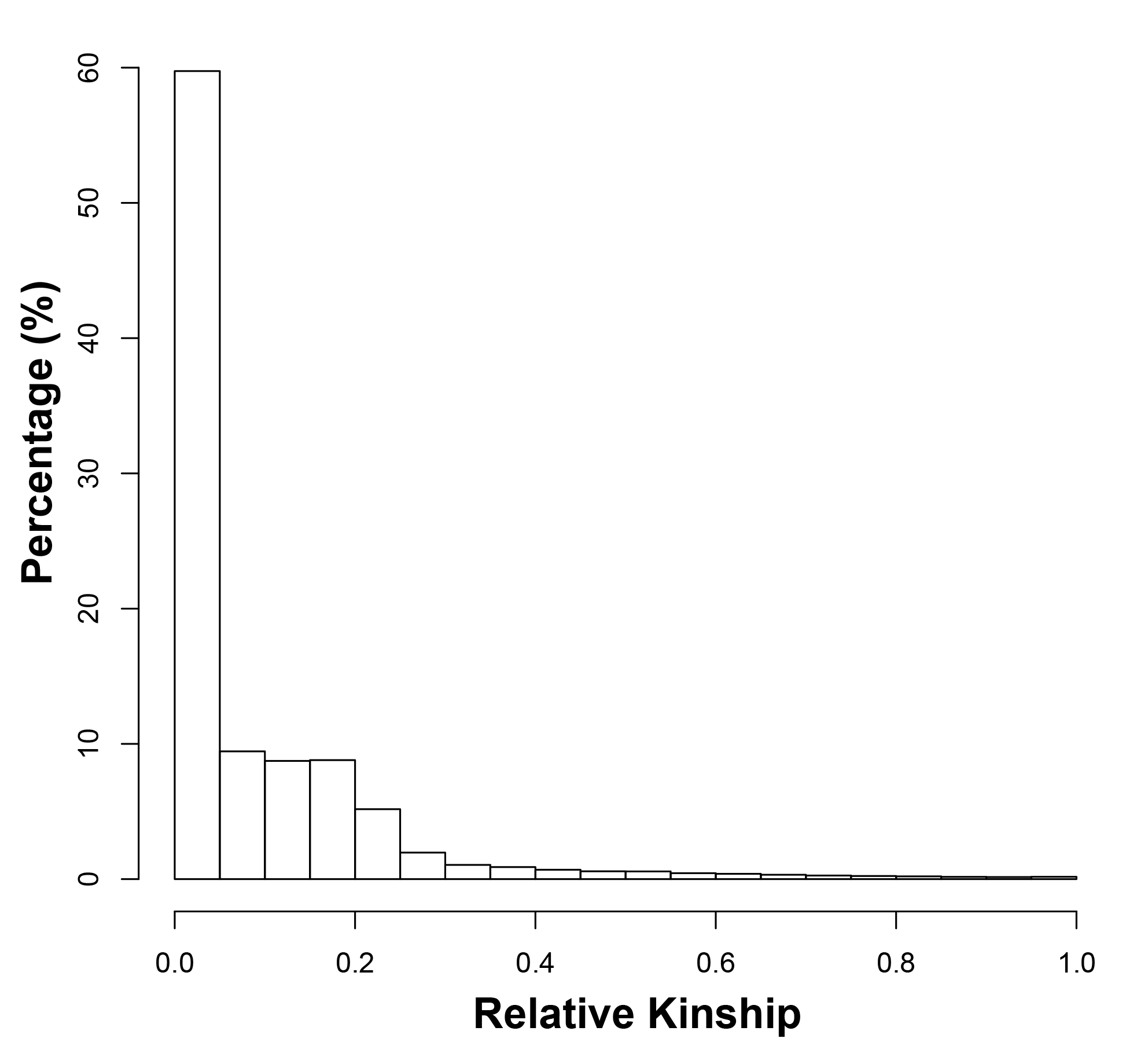

Supplement: Supplementary file 4 — High Resolution (TIFF 10884 kb) [file 11032_2017_622_MOESM2_ESM.tif]

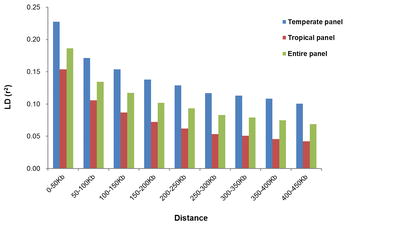

Supplement: Supplementary file 5 — Mean r2 of different physical distances for temperate, tropical and entire panels.(JPEG 10 kb) [file 11032_2017_622_Fig6_ESM.jpg]

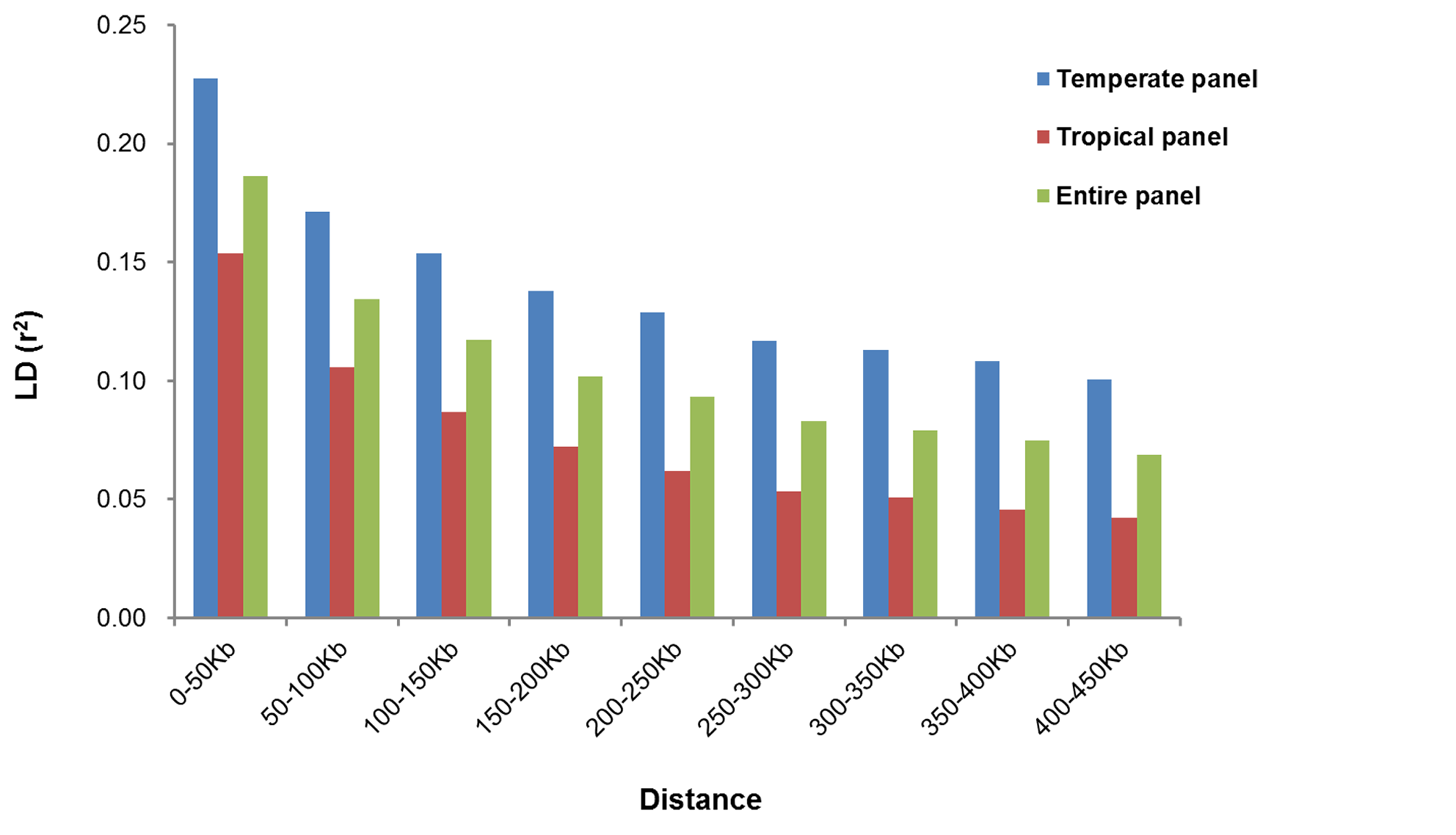

Supplement: Supplementary file 6 — High Resolution (TIFF 8715 kb) [file 11032_2017_622_MOESM3_ESM.tif]

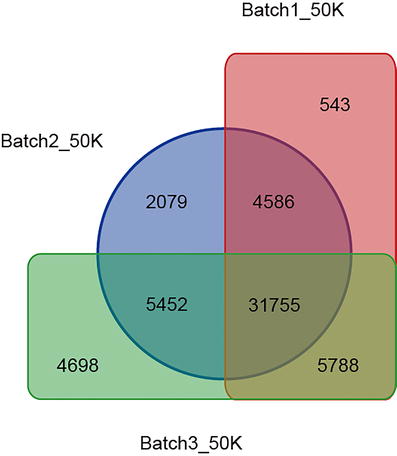

Supplement: Supplementary file 7 — Venn diagram summarizing the probe sets that were common in three independent runs of Illumina® MaizeSNP50 BeadChip. (JPEG 15 kb) [file 11032_2017_622_Fig7_ESM.jpg]

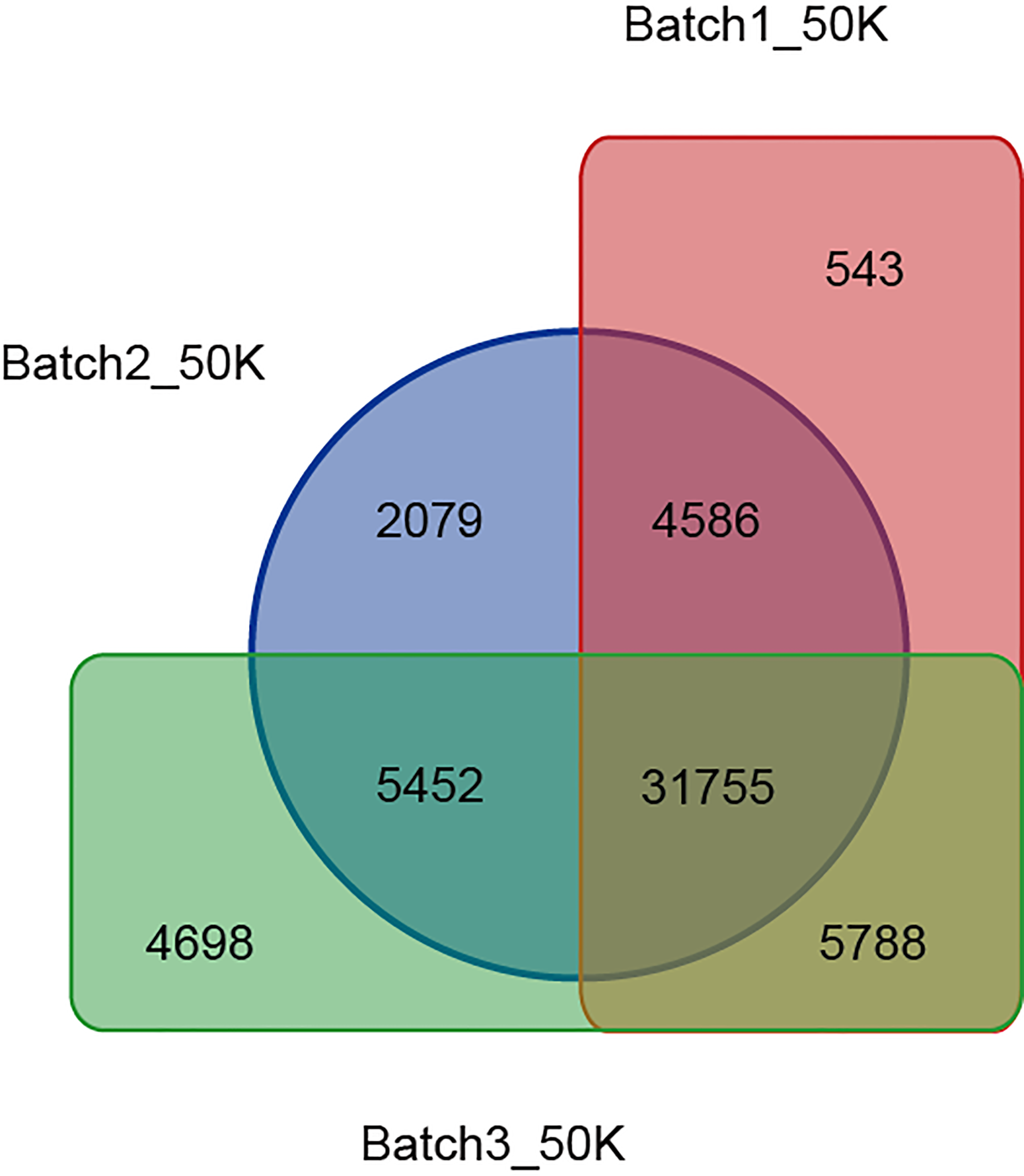

Supplement: Supplementary file 8 — High Resolution (TIFF 13268 kb) [file 11032_2017_622_MOESM4_ESM.tif]
